# Protocol: a multi-level intervention program to reduce stress in 9-1-1 telecommunicators

**DOI:** 10.1186/s12889-018-5471-0

**Published:** 2018-05-02

**Authors:** Hendrika Meischke, Michelle Lilly, Randal Beaton, Rebecca Calhoun, Ann Tu, Scott Stangenes, Ian Painter, Debra Revere, Janet Baseman

**Affiliations:** 1 Northwest Center for Public Health Practice, University of Washington, 1107 NE 45th St. Suite 400, Seattle, WA 98105 USA; 2 Department of Psychology, Psychology-Computer Science Building, Northern Illinois University, DeKalb, IL 60115 USA; 3 Department of Psychosocial and Community Health, University of Washington, Box 357263, Seattle, WA 98195 USA; 4 Center for Child and Family Well-Being, University of Washington, Box 351525, Seattle, WA 98195 USA; 5 OEM Program, University of Washington, Box 359739, 325 9th Ave, Seattle, WA 98104 USA; 6 Department of Health Services, University of Washington, Box 354809, Seattle, WA 98195 USA; 7 Department of Epidemiology, University of Washington, Box 354809, Seattle, WA 98195 USA

**Keywords:** 9-1-1 dispatcher, Emergency medical services, Mindfulness, Stress reduction

## Abstract

**Background:**

Nationwide, emergency response systems depend on 9-1-1 telecommunicators to prioritize, triage, and dispatch assistance to those in distress. 9-1-1 call center telecommunicators (TCs) are challenged by acute and chronic workplace stressors: tense interactions with citizen callers in crisis; overtime; shift-work; ever-changing technologies; and negative work culture, including co-worker conflict. This workforce is also subject to routine exposures to secondary traumatization while handling calls involving emergency situations and while making time urgent, high stake decisions over the phone. Our study aims to test the effectiveness of a multi-part intervention to reduce stress in 9-1-1 TCs through an online mindfulness training and a toolkit containing workplace stressor reduction resources.

**Methods/design:**

The study employs a randomized controlled trial design with three data collection points. The multi-part intervention includes an individual-level online mindfulness training and a call center-level organizational stress reduction toolkit. 160 TCs will be recruited from 9-1-1 call centers, complete a baseline survey at enrollment, and are randomly assigned to an intervention or a control group. Intervention group participants will start a 7-week online mindfulness training developed in-house and tailored to 9-1-1 TCs and their call center environment; control participants will be “waitlisted” and start the training after the study period ends. Following the intervention group’s completion of the mindfulness training, all participants complete a second survey. Next, the online toolkit with call-center wide stress reduction resources is made available to managers of all participating call centers. After 3 months, a third survey will be completed by all participants. The primary outcome is 9-1-1 TCs’ self-reported symptoms of stress at three time points as measured by the C-SOSI (Calgary Symptoms of Stress Inventory). Secondary outcomes will include: perceptions of social work environment (measured by metrics of social support and network conflict); mindfulness; and perceptions of social work environment and mindfulness as mediators of stress reduction.

**Discussion:**

This study will evaluate the effectiveness of an online mindfulness training and call center-wide stress reduction toolkit in reducing self-reported stress in 9-1-1 TCs. The results of this study will add to the growing body of research on worksite stress reduction programs.

**Trial registration:**

ClinicalTrials.gov Registration Number: NCT02961621 Registered on November 7, 2016 (retrospectively registered).

## Background

The effectiveness and efficiency of an emergency response system depends on 9-1-1 telecommunicators (TCs), the emergency call receivers and dispatchers who are the *first* first-responders to assist people in distress. 9-1-1 TCs prioritize and triage incoming calls delivered via myriad channels (cell and landline phone; TTY for deaf-and-hard-of-hearing citizens); collect and distill call information; dispatch police, firefighters, emergency medical technicians, and paramedics; and may issue medical and other instructions to callers while they await the assistance dispatched to the scene. Each 9-1-1 TC fields thousands of calls every year, ranging from trivial to life-threatening, while simultaneously monitoring and entering data into up to six different computer screen displays in real time during each call.

Much of the research on stress in first responders has been focused on police and firefighters, and the relationship between their trauma exposures and post-traumatic stress disorder (PTSD) [1, 2]. Although it is known that vicarious or secondary exposure can create secondary traumatic stress (i.e., behaviors and emotions resulting from knowledge about a traumatizing event that was experienced by another person and the desire to help that person) [3, 4] or compassion fatigue (i.e., cumulative stress) [4–7] in first responders, few studies have focused specifically on the occupational stressors and work-related stress symptoms of 9-1-1 TCs. Troxell (2008), one of the first to study 9-1-1 TCs, reported high peritraumatic distress (i.e., responses occurring at the time of a trauma and immediately after) and a positive relationship between peritraumatic distress and compassion fatigue in 9-1-1 TCs [8]. Pierce and Lilly (2012) added to these findings by assessing traumatic call exposure, peritraumatic distress, and PTSD symptoms in 9-1-1 TCs, reporting high levels of peritraumatic distress and a moderate, positive relationship between peritraumatic distress and PTSD symptom severity. This research suggests that direct, physical “incident scene” exposure(s) to traumata may not be necessary to cause or to increase risk for PTSD in 9-1-1 TCs [9].

The adverse outcomes associated with exposure to acute and chronic occupational stressors are costly and include physical and mental diseases, and impaired work performance, which can threaten the attainment of an organization’s critical mission(s) [10]. In addition to trauma exposures, 9-1-1 TCs are subject to those stressors commonly encountered by non-emergency call center workforces, including: fast-paced and time-urgent decision-making; tense interactions with distressed and sometimes abusive callers; time pressure to meet call processing requirements; and a negative work culture, which can include co-worker conflict, and a lack of appreciation or recognition from supervisors [11, 12]. Rameshbabu et al. (2013) reported that rotating shifts, common in 9-1-1 TCs, and their accompanying inadequate sleep were negatively associated with physical health outcomes in call center personnel [13]. Long hours of sedentary, high mental demand computer work, also ubiquitous in TCs, have been found to increase the risk for musculoskeletal symptoms [14] which can also be associated with psychological distress [15, 16]. Workplace environmental conditions such as lighting, ventilation, temperature, break/lunch room availability, and workstation ergonomics have also been found to exacerbate work-related stress in TC workforces [11, 12]. In addition, frequent technology updates including incremental and major “upgrades” in technologies, also common in the TC workplace, can cause “technostress” which has been shown in other worker groups to be associated with decreased job satisfaction and declines in productivity, commitment to the organization, and intention to remain on the job. [17]

### Project aims

The objective of this study is to develop and test the effectiveness of an evidence-based, multi-level intervention program designed to reduce stress in 9-1-1 TCs. The project’s primary aim is to:

- develop, implement, and test the effectiveness of an individual-level online mindfulness training to reduce stress in 9-1-1 telecommunicators.

The secondary aims are to:

- develop and test the effectiveness of an organizational-level online worksite stress reduction toolkit utilized by 9-1-1 TC managers that is designed to reduce or minimize sources of stress in the 9-1-1 call center environment.
- test if the multi-level intervention program will lead to increases in TCs’ positive perceptions of the call center social work environment.
- test if participation in the mindfulness training increases mindfulness.
- test if increases in TC’s positive perceptions of the call center social work environment and increases in mindfulness serve as mediators of stress reduction in 9-1-1 TCs.

### Conceptual framework

**Fig. 1:**
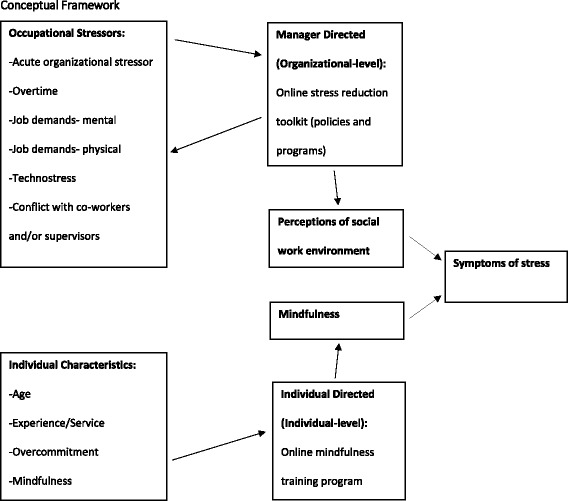
Conceptual Framework

Borrowing from the literature on occupational stress, individual stress, and coping response we developed a conceptual framework that describes how this multilevel intervention is expected to effect stress in 9-1-1 telecommunicators (Fig. 1). At the individual level, mindfulness training is hypothesized to increase an individual’s ability to regulate their emotional response to stressors, whereas the call center manager-directed organizational level intervention is hypothesized to reduce worksite stressors and increase perceived social support by implementation of targeted strategies and worksite policies.

### Mindfulness and stress

While there is no universally agreed upon definition, mindfulness can be defined as the individual’s ability to pay attention to and be aware of present moment experience, closely observe and describe sensations, perceptions, thoughts, and feelings, and act with full awareness [18]. A growing literature base has established the salutary effects of mindfulness-based interventions (MBIs). MBIs model, teach and cultivate the inner attentional resources of a trainee with the goal of learning to recognize and accept stress responses. Through recognition and acceptance, individuals learn to no longer rely on avoidance or suppression of emotional responses. Avoidance and suppression of emotional responses have been connected to greater stress levels and psychopathology following exposure to distressing events [19–23]. Mindfulness has been reported to be positively correlated with compassion satisfaction [24, 25] and inversely correlated with compassion fatigue [26–28] and burnout [29–31]. Mindfulness has also been associated with reduced stress in firefighters and paramedics [32] and MBIs have also been associated with fewer PTSD symptoms in combat veterans [33–38]. Specific to 9-1-1 TCs, a recent cross sectional/non-intervention study showed that increased mindfulness was inversely correlated with self-reported symptoms of stress [39]. And regarding delivery of mindfulness training, *online* MBIs have been shown to be effective in a number of workplace settings [40–46].

### Worksite environment and stress

In occupations such as the 9-1-1 TCs’, in which work demands are high and worker control is low, employee wellness and stress-mediated outcomes can impact attrition, turnover, burnout/exhaustion and absenteeism [47, 48]. Worksite characteristics affect employees’ productivity, satisfaction, and stress; however, an array of interventions may improve the quality of the work environment, and worksite health promotion activities can support employees’ healthy behaviors. The role of managers in particular, as a resource and support to employees has been highlighted as a significant component of organizational-level interventions that can reduce or mitigate stress [49–51]. In addition, interpersonal conflict between employees is a stressor amenable to intervention, for example, by training supervisors to adopt strategies for conflict management [52] and assisting organizations in adoption of anti-bullying policies [53]. Other known strategies for promoting organization-level wellness include healthy nutrition and exercise programs [54] and the reduction or elimination of environmental hazards in the workplace [11]. Thus, an array of interventions have been documented to improve the quality of the work environment, reduce symptoms of stress, and improve worker health- but the vast majority of this research has been conducted with non-9-1-1 TC workers.

## Methods/design

### Study design

**Fig. 2:**
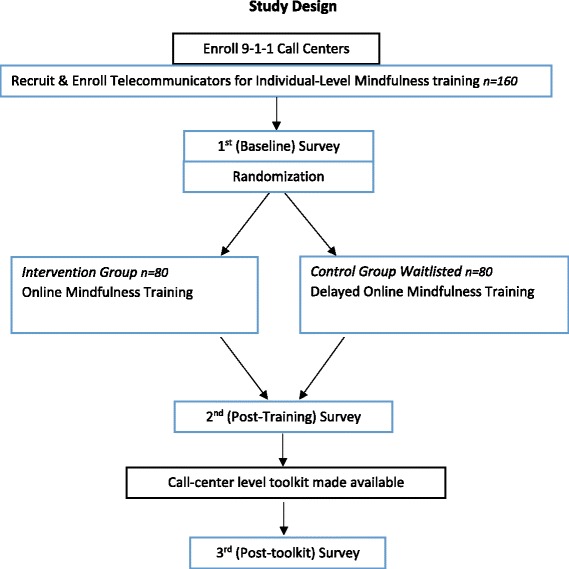
Study Design

The study is a longitudinal multi-stage study design that consists of a randomized controlled trial to evaluate the effectiveness of an online mindfulness training in reducing symptoms of stress in 9-1-1 call-center TCs, followed by 3 months access to an online call center stress reduction activity toolkit for the call center managers. We will recruit TCs from multiple enrolled 9-1-1 call centers nationwide. As shown in Fig. 2, at enrollment, TCs will complete a baseline stress survey and will then be randomized to either a mindfulness training intervention or wait-list control group. Following the mindfulness training, both the intervention and control groups will complete a post-training survey. Next, managers from all participating call centers will be provided access to the online Toolkit. After three months of access, all 9-1-1 TCs (training and control groups) will be asked to complete a post-toolkit survey.

### Population and procedures

The study population consists of 9-1-1 TCs working in multiple 9-1-1 call centers serving urban, suburban and rural areas in the United States. All participating call centers respond to 9-1-1 calls for fire, medical or police emergencies, with many centers responding to all three emergency call types.

Recruitment will be conducted in two stages. First, the study team will conduct an outreach campaign to 9-1-1 call centers, using industry publications and listserv announcements, to enroll call centers into the study. Next, at enrolled call centers, 9-1-1 TCs will be recruited through staff announcements, recruitment flyers, email, and word-of-mouth. After obtaining electronic informed consent, participants will be asked to provide demographic (age range, gender) and other information (length of employment, etc.) and complete the baseline survey. All participant information is stored on computers.

### Randomization

Respondents from each enrolled call center who are eligible, have provided their informed consent, and have completed the baseline survey, will be randomized into either the mindfulness training group or wait-list control group.

### Description, Design & Development of Interventions

#### Individual-level mindfulness training

The online mindfulness training is composed of seven modules to be completed weekly, over a 7-week time period. Each weekly online module is expected to require participants between 20 to 30 min to complete. Each individual module consists of the following components:

- A short video introducing the theme for the week
- A few paragraphs describing the theme and activities for the week
- An audio-guided meditative stress reduction exercise
- Suggestions for brief mindfulness activities that can be performed throughout the day
- A brief check-in form for participants to let study staff know how the training is going
- A moderated discussion board on which participants can post questions/comments if they wish

The mindfulness training is developed by clinicians and investigators who are trained mindfulness teachers and practitioners. The training is adapted from the widely-used Mindfulness-Based Stress Reduction (MBSR) program, an evidence-based training program originally implemented as in-person training [55, 56]. For our project, the MBSR training content was modified, abbreviated, and revised to accommodate an online format and tailored to meet the constraints and needs of the 9-1-1 TC population.

#### Organizational-level stress reduction toolkit

The online Toolkit is comprised of several modules providing evidence-based and expert-informed content and strategies for call center managers to consider and implement in an effort to reduce select stressors and increase network support in their worksites. The specific topics were selected based upon consultations and guidance from our research program advisory board consisting of call center managers from throughout our catchment area. The on-line toolkit includes modules focused on the following content areas: Conflict management; Bullying in the Workplace; and Reducing Technostress; with additional resources on Health and Wellness. Content is specifically tailored for managers and for their utility in 9-1-1 call centers.

### Procedures

#### Implementation protocols

After completing the baseline survey, participants will be assigned a study ID number and are randomized to either start the online mindfulness training (the intervention group) or are assigned to the wait-list control group.

Two weeks following the end of the online mindfulness training all participants will be asked to complete the second survey. Two weeks following the end of the call-center-level toolkit availability, all participants will be asked to complete the third survey.

#### Online mindfulness training

During the 7-week training period, the intervention group will be contacted each week, receiving two emails: one containing the link to that week’s training module and one providing additional suggestions for incorporating that week’s stress reduction activities into daily living. Participants will be encouraged to complete the training on a designated weekday to allow approximately 7 days between each module. Participants will also be encouraged to practice the mindfulness skills introduced in the module throughout the week for approximately 10 min every day.

#### Online stress reduction toolkit

Following the intervention group participants’ completion of the mindfulness training and completion of the second survey, managers of all participating call centers will be provided with a link to access the Toolkit and are asked to share the link with other administrative personnel at their call center who may be in a position to implement any of the suggested activities. Managers will have access to the Toolkit for 3 months and will receive bi-monthly emails encouraging them to use the Toolkit content and directing them to specific modules of the Toolkit. After 3 months access to the Toolkit, all participants complete the third survey.

### Measurements

#### Main outcome

The study main outcome is the TC’s self-reported stress as measured by the Calgary Symptoms of Stress Inventory (C-SOSI), a validated 56-item scale designed to assess various subjective symptoms of stress with 8 factor derived subscales, each consisting of 6-9 items: Depression, Anger, Muscle Tension, Cardiopulmonary Arousal, Sympathetic Arousal, Neurological/GI, Cognitive Disorganization, and Upper Respiratory Symptoms [57]. Subjects are asked to indicate the frequency with which they have experienced a particular symptom during the prior one week timeframe.

#### Mediating variables

Data regarding the following variables hypothesized to serve as mediators to the main outcome will be collected.

- *Social Support***:** Perceptions regarding social support (at work with coworkers and at home with family and friends), assessed by two separate visual analogue scales (VAS). Prior research has established the reliability and concurrent validity of these measures in another first responder sample [58].
- *Network Conflict*: The degree of conflict experienced at home and at work, assessed by two separate VAS. Prior research has established the reliability and concurrent validity of these measures in another first responder sample [58].
- *Mindfulness*: The Mindful Attention Awareness Scale (MAAS) will assess attentional sensitivity to psychological, somatic, and environmental cues [59]. Prior research has documented internal consistency reliability estimates were of good quality (α ranged = .89–.93) and test–retest reliability correlations between the repeated MAAS measures were all of medium-to-large magnitude and statistically significant [60]. The Five Facet Mindfulness Questionnaire (FFMQ) will assess five factors associated with mindfulness. Sound psychometric properties for the FFMQ have been consistently observed, including construct validity for the global FFMQ score, as well as adequate to strong internal consistency for the global score and subscales (α = .67-.93) [61–68]. The five factor hierarchical structure has been confirmed in samples of mediators and non-mediators [61, 68].

#### Covariates

We will collect the following data that may serve as covariates or confounders with our main outcome.

- *Demographics*: Participants are asked to provide their age, gender, and years of experience as 9-1-1 TCs.
- *Overcommitment (OC)*: OC refers to an individual’s exhaustive coping style which can adversely impact the health and well-being. [69, 70]. In our prior work, OC has been positively related to symptoms of stress and negatively related to mindfulness in a sample of 9-1-1 TCs [39]. OC items are drawn from the Effort-Reward Imbalance (ERI) scale used to measure occupational efforts relative to rewards [69]. We will add one item to increase its relevance to 9-1-1 TC work.
- *Overtime*: 9-1-1 TCs will be asked if they have worked overtime, and if it was voluntary or mandatory.
- *Acute stressor or stress event*: Call center managers will be asked if there was an unusual organization-wide acute stressful event during the study period.
- *Job Demands - mental*: Measured with the Mental Demands subscale (5 items) from the National Institutes for Occupational Safety and Health (NIOSH) Generic Job Stress Questionnaire. [71, 72]
- *Job Demands - physical*: Measured with the Job Requirements subscale (10 items) from the NIOSH Generic Job Stress Questionnaire. [71, 73]
- *Technostress*: Technostress is measured using items drawn from the Techno-uncertainty and Techno-insecurity sub-scales of the Technostress creators’ Scale [74] and we will add one Techno-insecurity item specific to 9-1-1 TC work.

### Sample size and power calculations

We anticipate randomizing a total of 160 9-1-1 TCs into two groups (~ 80 per group), and obtaining outcome data on survey measures from a minimum of 65 per group after accounting for attrition (allowing for a loss to follow-up of about 20%). A rough estimate of a clinically meaningful effect size was obtained after review of studies of mindfulness interventions that used the C-SOSI or SOSI measure as an outcome. We found seven studies [75–81] that reported sufficient information to estimate the effect size and within-participant standard deviations. These suggest that an effect size of 0.4 to 0.5 represents a clinically meaningful effect size. Based on our prior work measuring 9-1-1 TC stress utilizing the C-SOSI, this corresponds to a change in C-SOSI score of about 15 [39]. To give some context, in an unpublished analysis of stress in this population, a change in C-SOSI score of 15 was associated with a 30% increase in the use of sick days. Power calculations were performed using a two-sample t-test of the pre-post difference in C-SOSI scores, under the assumption of an effect size of 0.5, a type I error rate of 5%. To achieve 80% power requires data on 65 participants per group; allowing for a loss to follow up rate of 18% would require a total of 160 participants.

### Statistical analyses

The primary hypothesis for this intervention study is that TCs who are randomized to the online mindfulness training will report fewer symptoms of stress compared to TCs who are randomized to the wait-listed control group.

#### Primary analyses

To examine the primary hypothesis we will use repeated measures mixed effects models, with differences assessed by interaction terms between randomization group and follow-up time. The models will include fixed effects for group (2 levels) and time (3 levels; baseline, post intervention and follow up), site, time–by-treatment interaction, and an interaction term between the implementation of the center wide toolkit and the third time point, with a random intercept for participant. Differences between groups will be assessed using the time-by-treatment interaction term.

The primary outcome variable will be the C-SOSI symptoms of stress scale. Hypotheses will be conducted at the 0.05 level of significance without adjustment for multiple comparisons, with group assigned according to intent-to-treat.

The effects of possible differential drop-out rates between the two groups will be assessed using sensitivity analyses under the assumption that data is not missing at random. Multiple imputation will be used to impute missing values for the outcome variable under varying levels of bias, and the resulting association between level of bias and differences between the groups estimated.

#### Secondary analyses

The secondary outcomes examined will include mindfulness and perceptions of social work environment. We anticipate conducting the following analyses:

- *Effect of the intervention on mindfulness*: We will examine the effect of the intervention on mindfulness score using the same model as the primary analysis with MAAS score as the outcome.
- *Effect of intervention on perceptions of social work environment*: We will examine the effect of the intervention on perceptions of social work environment (social support and network conflict) using the same model as the primary analysis with the 4 VAS.
- *Analysis of mindfulness and perceptions of social work environments as mediators of stress reduction*: We will conduct analyses of the mediation effect of mindfulness (MAAS measures) and perceptions of social work environments (workplace social support and conflict VAS measures) on the intervention, estimating the direct and indirect causal effects using the approach of Imai [73].
- *Effect of training participation on stress:* Training participation will be examined using two measures: number of trainings completed as measured by access logs for viewing training videos and self-report mean number of days per week on which mindfulness was practiced. The effects of level of participation will be examined using repeated measures mixed effects models, with the effect of participation level assessed by interaction terms between level of participation and follow-up time. The models will include level of participation as a covariate, fixed effect for time (3 levels; baseline, post intervention and follow up), site, time–by-level-of-participation interaction and a random intercept for participant.
- *Effect of online Toolkit*: A secondary aim of this study is to determine the extent to which an online Toolkit for reducing occupational stress in call centers improves call center climate four months post implementation of the Toolkit. We will use a multi-level model to examine the effects of implementation of the toolkit (as measured through the manager survey) on mean call center stress levels and call center climate, as measured by workplace support and conflict measures.

## Discussion

Emergency response systems depend on 9-1-1 telecommunicators, the emergency call receivers and dispatchers who are the *first* first-responders to assist callers contacting 9-1-1 for aid in emergencies. Research shows that this workforce is exposed to acute and chronic occupational stressors; both trauma exposures specific to emergency response, and those stressors commonly encountered by non-emergency call center workforces. This study will evaluate if an online mindfulness training and call center-wide stress reduction resources can reduce stress in 9-1-1 TCs. The results of this study will add to the growing body of research on worksite stress reduction programs, the use of online mindfulness training in workforce stress reduction, and stress reduction in 9-1-1 telecommunicators.

## Abbreviations

C-SOSI: Calgary Symptoms of Stress Inventory
ERI: Effort-Reward Imbalance
FFMQ: Five Facet Mindfulness Questionnaire
MAAS: Mindfulness Attention Awareness Scale
MBI: Mindfulness-Based Intervention
MBSR: Mindfulness-Based Stress Reduction
NIOSH: National Institute for Occupational Safety and Health
OC: Overcommitment
PTSD: Post-Traumatic Stress Disorder
SOSI: Symptoms of Stress Inventory
TC: Telecommunicator
VAS: Visual Analogue Scale

## References

[1] Corneil W, Beaton R, Murphy S, Johnson C, Pike K (1999). Exposure to traumatic incidents and prevalence of posttraumatic stress symptomatology in urban firefighters in two countries. J Occup Health Psychol.

[2] Marmar CR, McCaslin SE, Metzler TJ, Best S, Weiss DS, Fagan J (2006). Predictors of posttraumatic stress in police and other first responders. Ann N Y Acad Sci.

[3] Cieslak R, Shoji K, Douglas A, Melville E, Luszczynska A, Benight CC (2014). A meta-analysis of the relationship between job burnout and secondary traumatic stress among workers with indirect exposure to trauma. Psychol Serv.

[4] Newell JM, MacNeil GA (2010). Professional burnout, vicarious trauma, secondary traumatic stress, and compassion fatigue: a review of theoretical terms, risk factors, and preventive methods for clinicians and researchers. Best Pract Ment Health.

[5] Kanno H, Giddings MM (2017). Hidden trauma victims: understanding and preventing traumatic stress in mental health professionals. Soc Work Ment Health.

[6] Knight C (2010). Indirect trauma in the field practicum: secondary traumatic stress, vicarious trauma, and compassion fatigue among social work students and their field instructors. J Baccalaureate Soc Work.

[7] Nader K (2008). Review of help for the helper: the psychophysiology of compassion fatigue and vicarious trauma. Traumatology.

[8] Troxell, RM. Indirect exposure to the trauma of others: the experiences of 9-1-1 telecommunicators (Doctoral dissertation). Retrieved from ProQuest Dissertations and Theses database. 2008. Pub no: 3335425.

[9] Pierce H, Lilly MM (2012). Duty-related trauma exposure in 911 telecommunicators: considering the risk for posttraumatic stress. J Trauma Stress.

[10] O'Keefe LC, Brown KC, Christian BJ (2014). Policy perspectives on occupational stress. Workplace Health Saf.

[11] Wiegand DM. Evaluation of job stress and work-related health concerns of a telephone call center. NIOSH Report # 2012-0211-3197. US DHHS, CDC, NIOSH. 2013. http://www.cdc.gov/niosh/hhe/reports/pdfs/2012-0211-3197.pdf. Accessed 30 Nov 2017.

[12] Driscoll RJ, Tubbs RL, Habes DJ. NIOSH Health Hazard Evaluation Report. NIOSH Report # 97-0137-3026. US DHHS, CDC, NIOSH 2007. https://www.cdc.gov/niosh/hhe/reports/pdfs/1997-0137-3026.pdf. Accessed 30 Nov 2017.

[13] Rameshbabu A, Reddy DM, Fleming R (2013). Correlates of negative physical health in call center shift workers. Appl Ergon.

[14] Griffiths KL, Mackey MG, Adamson BJ (2007). The impact of a computerized work environment on professional occupational groups and behavioural and physiological risk factors for musculoskeletal symptoms: a literature review. J Occup Rehabil.

[15] Croidieu S, Charbotel B, Vohito M, Rebnaud L, Jaussaud J, Bourboul C (2008). Call-handler’s working conditions and their subjective experience of work: a transversal study. Int Arch Occup Environ Health.

[16] Charbotel B, Croidieu S, Vohito M, Guerin AC, Renaud L, Jaussaud J (2009). Working conditions in call-centers, the impact on employee health: a transversal study. Part II. Int Arch Occup Environ Health.

[17] Tarafdar M, Tu Q, Ragu-Nathan BS, Ragu-Nathan TS (2007). The impact of technostress on role stress and productivity. J MIS.

[18] Kabat-Zinn J (2003). Mindfulness-based interventions in context: past, present, and future. Clin Psychol Sci Pract.

[19] Briere J, Hodges M, Godbout N (2010). Traumatic stress, affect dysregulation, and dysfunctional avoidance: a structural equation model. J Trauma Stress.

[20] Hayes SC, Wilson KG, Gifford EV, Follette VM, Strosahl K (1996). Experiential avoidance and behavioral disorders: a functional dimensional approach to diagnosis and treatment. J Consult Clin Psychol.

[21] Kumpula MJ, Orcutt HK, Bardeen JR, Varkovitzky RL (2011). Peritraumatic dissociation and experiential avoidance as prospective predictors of posttraumatic stress symptoms. J Abnorm Psychol.

[22] Moore SA, Zoellner LA, Mollenholt N (2008). Are expressive suppression and cognitive reappraisal associated with stress-related symptoms?. Behav Res Ther.

[23] Nitzan-Assayag Y, Yuval K, Tanay G, Aderka IM, Vujanovic AA, Litz B (2017). Reduced reactivity to and suppression of thoughts mediate the effects of mindfulness training on recovery outcomes following exposure to potentially traumatic stress. Mindfulness.

[24] Gregory A (2015). Yoga and mindfulness program: the effects on compassion fatigue and compassion satisfaction in social workers. J Relig Spiritual Social Work.

[25] Thomas JT, Otis MD (2010). Intrapsychic correlates of professional quality of life: mindfulness, empathy, and emotional separation. J Soc Soc Work Res.

[26] Brown JLC, Ong J, Mathers JM, Decker JT (2017). Compassion fatigue and mindfulness: comparing mental health professionals and MSW student interns. J Evid Inform Soc Work.

[27] Duarte J, Pinto-Gouveia J (2016). Effectiveness of a mindfulness-based intervention on oncology nurses' burnout and compassion fatigue symptoms: a non-randomized study. Int J Nurs Stud.

[28] Lomas T, Medina JC, Ivtzan I, Rupprecht S, Hart R, Eiroa-Orosa FJ (2017). The impact of mindfulness on well-being and performance in the workplace: an inclusive systematic review of the empirical literature. Eur J Work Organ Psychol.

[29] Felton TM, Coates L, Christopher JC (2015). Impact of mindfulness training on counseling students' perceptions of stress. Mindfulness.

[30] Kaplan JB, Bergman AL, Christopher M, Bowen S (2017). Hunsinger, M. Role of resilience in mindfulness training for first responders. Mindfulness.

[31] Thompson IA, Amatea ES, Thompson ES (2014). Personal and contextual predictors of mental health counselors' compassion fatigue and burnout. J Ment Health Couns.

[32] Smith BW, Ortiz JA, Steffen LE, Tooley EM, Wiggins KT, Yeater EA, Montoya JD, Bernard ML (2011). Mindfulness is associated with fewer PTSD symptoms, depressive symptoms, physical symptoms, and alcohol problems in urban firefighters. J Consult Clin Psychol.

[33] Colgan DD, Christopher M, Michael P, Wahbeh H (2016). The body scan and mindful breathing among veterans with PTSD: type of intervention moderates the relationship between changes in mindfulness and post-treatment depression. Mindfulness.

[34] Felleman BI, Stewart DG, Simpson TL, Heppner PS, Kearney DJ (2016). Predictors of depression and PTSD treatment response among veterans participating in mindfulness-based stress reduction. Mindfulness.

[35] Kearney DJ, McDermott K, Malte C, Martinez M, Simpson TL (2013). Effects of participation in a mindfulness program for veterans with posttraumatic stress disorder: a randomized controlled pilot study. J Clin Psychol.

[36] King AP, Erickson TM, Giardino ND, Favorite T, Rauch SA, Robinson E (2013). A pilot study of group mindfulness-based cognitive therapy (MBCT) for combat veterans with posttraumatic stress disorder (PTSD). Depress and Anxiety.

[37] Niles BL, Klunk-Gillis J, Ryngala DJ, Silberbogen AK, Paysnick A, Wolf EJ (2012). Comparing mindfulness and psychoeducation treatments for combat-related PTSD using a telehealth approach. Psychol Trauma Theory Res Pract Policy.

[38] Possemato K, Bergen-Cico D, Treatman S, Allen C, Wade M, Pigeon W (2016). A randomized clinical trial of primary care brief mindfulness training for veterans with PTSD. J Clin Psychol.

[39] Meischke H, Painter I, Lilly M, Beaton R, Revere D, Calhoun B (2015). An exploration of sources, symptoms, and buffers of occupational stress in 9-1-1 emergency call centers. Ann Emerg Dispatch Response.

[40] Aikens KA, Astin J, Pelletier KR, Levanovich K, Baase CM, Park YY, Bodnar CM (2014). Mindfulness goes to work: impact of an online workplace intervention. J Occup Environ Med.

[41] Feicht T, Wittmann M, Jose G, Mock A, von Hirschhausen E, Esch T. Evaluation of a seven-week web-based happiness training to improve psychological well-being, reduce stress, and enhance mindfulness and flourishing: a randomized controlled occupational health study. Evid Based Complement Alternat Med. 2013; 10.1155/2013/676953. 10.1155/2013/676953 PMC3893772 24489588

[42] Krusche A, Cyhlarova E, Williams JMG (2013). Mindfulness online: an evaluation of the feasibility of a web-based mindfulness course for stress, anxiety and depression. BMJ Open.

[43] Michel A, Bosch C, Rexroth M (2014). Mindfulness as a cognitive–emotional segmentation strategy: an intervention promoting work–life balance. J Occup Organ Psychol.

[44] Morledge TJ, Allexandre D, Fox E, Fu AZ, Higashi MK, Kruzikas DT (2013). Feasibility of an online mindfulness program for stress management--a randomized, controlled trial. Ann Behav Med.

[45] MPJ S, WTM P, Bohlmeijer ET. Effectiveness of online mindfulness-based interventions in improving mental health: a review and meta-analysis of randomised controlled trials. Clin Psychol Rev, 2016. 45:102–14. 10.1016/j.cpr.2016.03.009. 10.1016/j.cpr.2016.03.009 27111302

[46] Wolever RQ, Bobinet KJ, McCabe K, Mackenzie ER, Fekete E, Kusnick CA (2012). Effective and viable mind-body stress reduction in the workplace: a randomized controlled trial. J Occup Health Psychol.

[47] Karasek R, Theorell T. Healthy work: stress, productivity, and the reconstruction of working life. New York: Basic Books; 1980.

[48] Xanthopoulou D, Bakker AB, Demerouti E, Schaufeli WB (2007). The role of personal resources in the job demands-resources model. Int J Stress Manag.

[49] Karasek RA (1979). Job demands, job decision latitude and mental strain: implications for job redesign. Admin Sci Q.

[50] Bakker AB, Demerouti E, Euwema MC (2005). Job resources buffer the impact of job demands on burnout. J Occup Health Psychol.

[51] Swider BW, Zimmerman RD (2010). Born to burnout: a meta-analytic path model of personality, job burnout, and work outcomes. J Voc Behav.

[52] Almost J, Wolff AC, Stewart-Pyne A, McCormick LG, Strachan D, D'Souza C (2016). Managing and mitigating conflict in healthcare teams: an integrative review. J Adv Nurs.

[53] Stagg SJ, Sheridan D (2010). Effectiveness of bullying and violence prevention programs. AAOHN J.

[54] McCoy K, Stinson K, Scott K, Tenney L, Newman LS (2014). Health promotion in small business: a systematic review of factors influencing adoption and effectiveness of worksite wellness programs. J Occup Environ Med.

[55] Kabat-Zinn J, Lipworth L, Burney R (1985). The clinical use of mindfulness meditation for the self-regulation of chronic pain. J Behav Med.

[56] Kabat-Zinn J (1990). Full catastrophe living: using the wisdom of your body and mind to face stress, pain, and illness.

[57] Carlson LE, Thomas BC (2007). Development of the Calgary symptoms of stress inventory (C-SOSI). Int J Behav Med.

[58] Beaton RD, Murphy SA, Pike KC, Corneil W (1997). Social support and network conflict in firefighters and paramedics. West J Nurs Res.

[59] Brown KW, Ryan RM (2003). The benefits of being present: mindfulness and its role in psychological well-being. J Pers Soc Psychol.

[60] Black DS, Sussman S, Johnson CA, Milam J (2012). Psychometric assessment of the mindful attention awareness scale (MAAS) among Chinese adolescents. Assessment.

[61] Baer RA, Smith GT, Hopkins J, Krietemeyer J, Toney L (2006). Using self-report assessment methods to explore facets of mindfulness. Assessment.

[62] Barnes SM, Lynn SJ (2010). Mindfulness skills and depressive symptoms: a longitudinal study. Imagination, Cognition Personality.

[63] Cash M, Whittingham K (2010). What facets of mindfulness contribute to psychological well-being and depressive, anxious, and stress-related symptomatology?. Mindfulness.

[64] Fisak B, von Lehe AC (2012). The relation between the five facets of mindfulness and worry in a non-clinical sample. Mindfulness.

[65] Hollis-Walker L, Colosimo K (2011). Mindfulness, self-compassion, and happiness in non-meditators: a theoretical and empirical examination. Pers Indiv Differ.

[66] Lavender JM, Gratz KL, Tull MT (2011). Exploring the relationship between facets of mindfulness and eating pathology in women. Cogn Behav Ther.

[67] Park T, Reilly-Spong M, Gross CR (2013). Mindfulness: a systematic review of instruments to measure an emergent patient-reported outcome (PRO). Qual Life Res.

[68] Baer RA, Smith GT, Lykins E, Button D, Krietemeyer J, Sauer S (2008). Construct validity of the five facet mindfulness questionnaire in meditating and nonmeditating samples. Assessment.

[69] Lau B. Effort-reward imbalance and overcommitment in employees in a Norwegian municipality: a cross sectional study. J Occup Med Toxicol. 2008;3(9) 10.1186/1745-6673-3-9. 10.1186/1745-6673-3-9 PMC2405796 18447923

[70] de Jonge J, Bosma H, Peter R, Siegrist J (2000). Job strain, effort-reward imbalance and employee well-being: a large-scale cross-sectional study. Soc Sci Med.

[71] Generic Job Stress Questionnaire. US DHHS, CDC, NIOSH. https://www.cdc.gov/niosh/topics/workorg/tools/pdfs/niosh-generic-job-stress-questionaire.pdf. Accessed 30 Nov 2017.

[72] Rationale for NIOSH Generic Job Stress Questionnaire. US DHHS, CDC, NIOSH. https://www.cdc.gov/niosh/topics/workorg/tools/pdfs/Rationale-For-Niosh-Generic-Job-Stress-Questionnaire.pdf

[73] Caplan RD, Cobb S, French JR, Harrison RV, and Pinneau SR. Job Demands and Worker Health. US DHHS CDC NIOSH. DHEW (NIOSH) Publication No. 75-160. 1975;1–353.

[74] Ragu-Nathan TS, Tarafdar M, Ragu-Nathan BS, Tu Q (2008). The consequences of technostress for end users in organizations: conceptual development and empirical validation. Inform Sys Res.

[75] Carlson LE, Speca M, Patel KD, Goodey E (2003). Mindfulness-based stress reduction in relation to quality of life, mood, symptoms of stress, and immune parameters in breast and prostate cancer outpatients. Psychosom Med.

[76] Carlson LE, Garland SN (2005). Impact of mindfulness-based stress reduction (MBSR) on sleep, mood, stress and fatigue symptoms in cancer outpatients. Int J Behav Med..

[77] Garland EL, Manusov EG, Froeliger B, Kelly A, Williams JM, Howard MO (2014). Mindfulness-oriented recovery enhancement for chronic pain and prescription opioid misuse: results from an early-stage randomized controlled trial. J Consul and Clin Psychol.

[78] Matchim Y, Armer JM, Stewart BR (2011). Effects of mindfulness-based stress reduction (MBSR) on health among breast cancer survivors. West J Nurs Res.

[79] Lerman R, Jarski R, Rea H, Gellish R, Vicini F (2012). Improving symptoms and quality of life of female cancer survivors: a randomized controlled study. Ann Surg Oncol.

[80] Zernicke KA, Campbell TS, Blustein PK, Fung TS, Johnson JA, Bacon SL (2013). Mindfulness-based stress reduction for the treatment of irritable bowel syndrome symptoms: a randomized wait-list controlled trial. Int J Behav Med.

[81] Zernicke KA, Campbell TS, Speca M, McCabe-Ruff K, Flowers S, Carlson LE (2014). A randomized wait-list controlled trial of feasibility and efficacy of an online mindfulness-based cancer recovery program: the eTherapy for cancer applying mindfulness trial. Psychosom Med.

